# Preoperative predictors of difficult hypopharyngeal exposure by retractor for transoral robotic surgery

**DOI:** 10.1007/s10147-018-1335-y

**Published:** 2018-08-13

**Authors:** Kazunori Fujiwara, Satoshi Koyama, Ryouhei Donishi, Takahiro Fukuhara, Naritomo Miyake, Hiromi Takeuchi

**Affiliations:** Department of Otolaryngology, Head and Neck Surgery, Faculty of Medicine, Tottori University, Nishimachi 36-1, Yonago, 683-8504 Japan

**Keywords:** Transoral surgery, Hypopharyngeal exposure, Parameter

## Abstract

**Introduction:**

Transoral endoscopic surgeries provide excellent oncologic outcomes while preserving speech and swallowing ability. However, feasibility has been a major concern about transoral surgery. Therefore, ensuring visualization of the surgical field and sufficient working space is required. The aim of this study was to evaluate the parameters in the preoperative assessment that affect hypopharyngeal exposure.

**Methods:**

Before transoral surgery, parameters regarding the patient’s neck and face such as modified Mallampati index, thyroid–mental distance (TMD), and ability to fully open the mouth were evaluated. Cephalometry and cervical spine radiography were performed preoperatively to evaluate the size of the mandible bone, mouth opening, and cervical spine extension. Mandibular bone parameters such as intergonion distance, mental–gonion distance, articulare–gonion distance, and aperture angle were measured. According to hypopharyngeal exposure using FKWO retractor, patients were divided into difficult hypopharyngeal exposure group (DHE) and non-difficult hypopharyngeal exposure group (non-DHE). Parameters were enrolled to evaluate the relationship between these parameters and DHE status.

**Results:**

This study included 51 patients, 37 in the non-DHE group and 14 in the DHE group. On radiographic evaluation, there was a significant difference in the degree of cervical lordosis between non-DHE and DHE patients. A significantly higher proportion of DHE patients had a history of radiotherapy compared with non-DHE patients.

**Conclusion:**

Patients with limited cervical extension and a history of previous radiotherapy might have difficult hypopharyngeal exposure during transoral surgery. This is the first report to suggest a classification system for hypopharyngeal exposure during transoral surgery.

## Introduction

Compared with radiotherapy, transoral surgery is a more minimally invasive treatment for laryngopharyngeal cancer. Advantages of the transoral approach include no skin incision, limited tissue dissection, and less disruption of speech and swallowing muscles, blood loss, damage to major neurovascular structures, and injury of normal tissue [1].

Since the 1990s, transoral laser microsurgery (TLM) has been used as an organ-preserving strategy with good oncological control and functional results, but it has not been widely used because of its technical difficulty. Many studies have reported the feasibility of transoral robotic surgery (TORS) for the treatment of laryngeal, hypopharyngeal, and supraglottic cancer with excellent results in terms of oncologic radicality and functional preservation [2, 3]. Therefore, recently, transoral robotic surgery has been gaining popularity as a new treatment modality for laryngopharyngeal cancer, and it is being performed widely [4].

Alternative transoral surgical methods have been developed, such as transoral videolaryngoscopic surgery (TOVS) [5, 6] and endoscopic laryngopharyngeal surgery (ELPS) [7]. In TORS and TOVS, a straight plate tongue depressor, such as the FKWO retractor (Olympus; Tokyo, Japan) is used to obtain laryngopharyngeal exposure and it also serves as a straight instrument. On the other hand, in ELPS, a curved laryngoscope is used. Since the surgical tools for TOVS are straight, this system is relatively easy to handle for general otolaryngologists. An additional advantage of TOVS is that the surgeon receives tactile sensations directly through the straight forceps and electrocautery instrument. Tactile sensation is very important when assessing tumor infiltration of surrounding structures.

The FKWO retractor is placed through the oral cavity and pharynx. The larynx and pharynx are almost exposed under general anesthesia without difficulty [8]. In some patients, the entire hypopharynx is not visualized with straight tools such as the FKWO retractor. In this situation, inadequate hypopharyngeal exposure may lead to incomplete tumor resection.

Several parameters that could predict difficult laryngeal exposure (DLE) have been investigated [8–10]. Short neck, stiff and muscular neck, macroglossia, retrognathia, obesity, and limited cervical spine extension have been suggested as possible causes of DLE based on cases of difficult endotracheal intubation, obstructive sleep apnea syndrome, and macrolaryngeal surgery [11–14].

Transoral surgery should be performed with sufficient exposure of the hypopharynx; however, predictors of difficult hypopharyngeal exposure in transoral surgery have not been reported. The aim of this study was to evaluate which parameters included as part of preoperative assessment affect hypopharyngeal exposure.

## Methods

### Patients

Between April 2015 and March 2017, 51 patients underwent transoral surgery in the Tottori University Minimum Invasive Surgical Center. Table 1 shows the distribution of conditions.

**Table 1:** Primary diseases of patients

Disease	
Cancer	40
Hypopharyngeal cancer	27
Oropharyngeal cancer	11
Supraglottic cancer	1
Cervical esophagus cancer	1
Benign tumor	6
Oropharyngeal tumor	4
Hypopharyngeal tumor	2
Non-tumor	5
Cricopharyngeal dysfunction	5

Preoperatively, all patients underwent endoscopic examination; computed tomography of the throat, neck, and chest with contrast; ultrasonography; and videofluoroscopy. For this study, we selected patients with the following characteristics: (1) age 20 years or older; (2) ECOG performance status of 0 or 1; (3) diagnosis of oropharyngeal, hypopharyngeal, supraglottic, or cervical esophageal squamous cell carcinoma, classified preoperatively as Tis, T1, or T2 and N0, N1, N1a, or N2b according to the 2010 UICC classification system, or diagnosis of cricopharyngeal dysfunction due to neurologic, iatrogenic, or inflammatory causes.

### Evaluated parameters

Before surgery, the dimensions of the patient’s neck and face [e.g., modified Mallampati index, thyroid–mental distance (TMD), and full mouth opening] were evaluated as well as general parameters such as age, weight, height, and body mass index (BMI). The modified Mallampati index was evaluated by visualization of oropharyngeal structures with the patient’s mouth open and tongue fully protruding. The modified Mallampati index was used to grade the degree to which the oropharynx was obscured as follows: grade 1, clearly visible tonsils, pillars, and soft palate; grade 2, only uvula, pillars, and the upper pole of tonsils are visible; grade 3, partially visible soft palate; and grade 4, only hard palate is visible [13]. The thyroid–mental distance was measured in centimeters from the mental prominence to the thyroid notch [15]. Full mouth opening refers to the distance between the tip of the upper and lower incisors in centimeters with the mouth fully open. BMI was calculated as weight (kilograms) divided by height squared (m^2^). Before surgery, we assessed whether the patient had a history of neck radiotherapy and cervical vertebral syndrome.

**Fig. 1:**
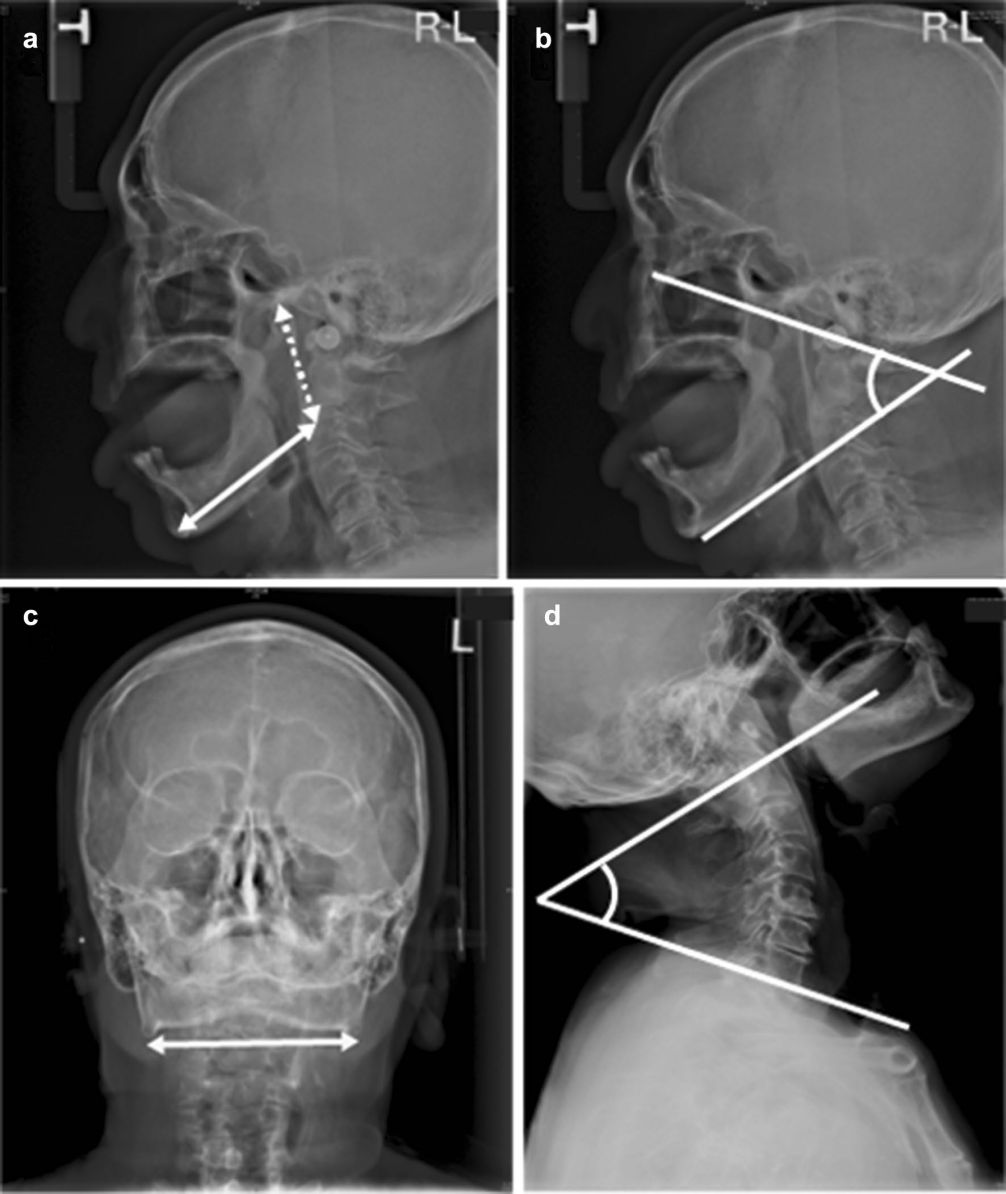
The cephalometry and cervical spine radiograpy. Mandibular bone parameters were measured by the cephalometry, such as mental–gonion distance (**a**; solid line), articulare–gonion distance (**a**; dot line), aperture angle (**b**), and intergonion distance (**c**; solid line). Cervical lordosis degree was measured by cervical spine radiography (**d**)

Cephalometry and cervical spine radiography were performed preoperatively to evaluate the size of the mandible bone, mouth opening, and cervical spine extension. The following mandible bone parameters were measured using the methods illustrated in Fig. 1: intergonion distance, mental–gonion distance, and articulare–gonion distance. Gonion was defined as the point at the intersection of posterior and inferior plane of the mandible bone. Articulare was defined as the junction between the inferior surface of the base of the skull and the posterior border of the ascending rami of the mandible. Menton was defined as the lowest point on the mandibular symphysis. In addition, we measured the aperture angle using the method illustrated in Fig. 1. Aperture angle was defined as the angle of the intersection of the inferior plane of the mandible bone and the plane connecting points on the orbital floor and the external auditory canal. Furthermore, we measured the degree of lordosis to evaluate cervical spine extension. Cervical lordosis angle was defined as the angle of the intersection of the superior plane of first cervical vertebra and the inferior plane of the seventh vertebral body.

### Intraoperative evaluation

Transoral surgery was performed under general anesthesia and muscle relaxation. Patients were intubated with an endotracheal tube (6.5 or 7.0 mm in diameter) and assessed in the sniffing position. Next, patients were positioned with full extension of the head and neck. A FKWO retractor was inserted to expose the hypopharynx and a flexible endoscope with angulation in 4 directions (Visera LTF-type VP; Olympus) used to visualize the hypopharynx.

**Fig. 2:**
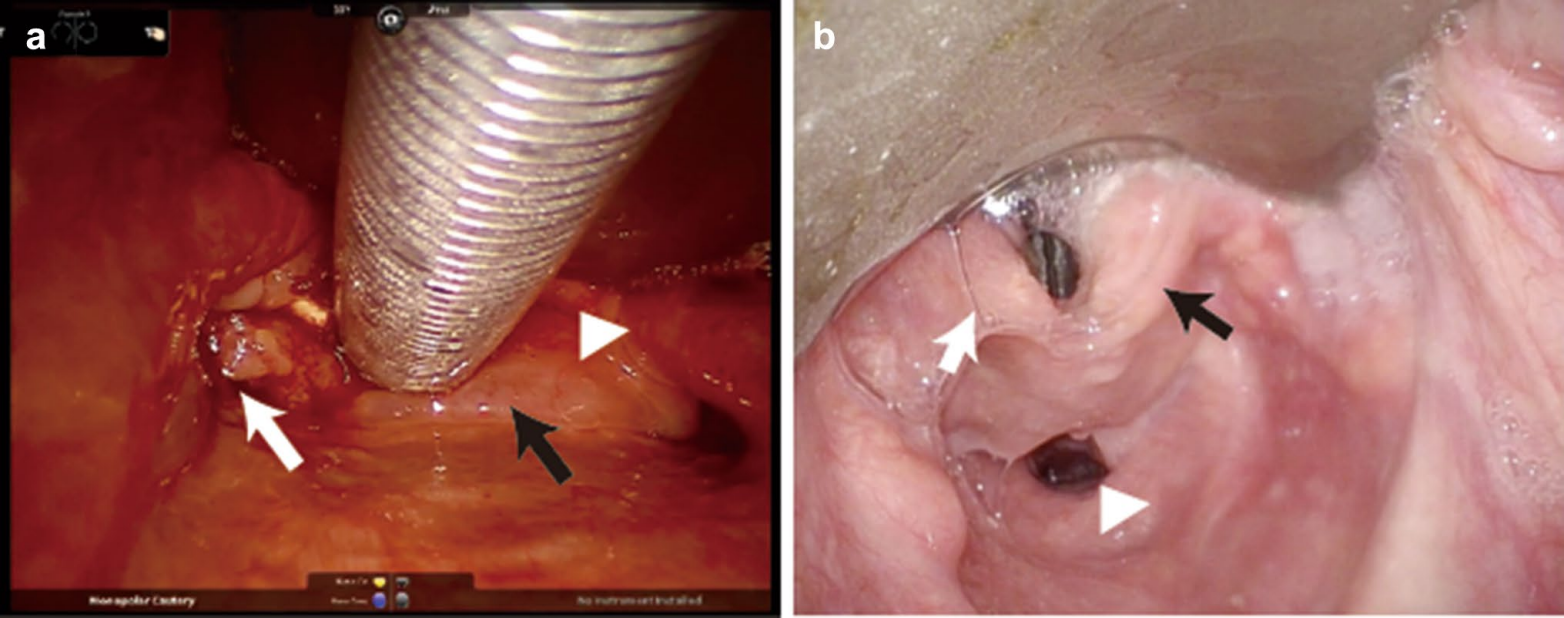
Hypopharyngeal exposure using FKWO retractor. Limited vision of pyriform sinus (difficult hypopharyngeal exposure: **a**). Right arytenoid (black arrow), hypopharyngeal carcinoma (white arrow), epiglottis (white triangle). Full vision of pyriform sinus (non-difficult hypopharyngeal exposure: **b**). Right arytenoid (black arrow), left arytenoid (white arrow), esophageal orifice (white triangle)

Patients were divided into two groups according by hypopharyngeal view. Patients with insufficient exposure of the hypopharynx to perform transoral surgery were classified into the difficult hypopharyngeal exposure group. The others were classified into the non-difficult hypopharyngeal exposure group (Fig. 2). Insufficient exposure was defined as a limited vision of supraglottic region and folded pyriform sinus. On the other hand, sufficient exposure was defined as full vision of the supraglottic region and an unfolded pyriform sinus as De Virgilio et al. [16].

For oropharyngeal, hypopharyngeal, cervical esophageal, and supraglottic cancer, TOVS and TORS were performed. The surgical method was based on patient preference. If hypopharyngeal exposure was inadequate using FKWO retractor, the patients were classified as DHE and ELPS was performed using a curved laryngoscope. If there was inadequate hypopharyngeal exposure with a curved laryngoscope, we selected alternative treatments such as chemoradiotherapy. For oropharyngeal and hypopharyngeal benign tumor, TOVS was performed. For cricopharyngeal dysfunction, endoscopic cricopharyngeal myotomy was performed. If hypopharyngeal exposure was inadequate, the patients were classified as DHE and transcervical cricopharyngeal myotomy was performed.

All patients gave informed consent. This study was approved by the Tottori University institutional review board (IRB number 2723).

### Statistical analysis

All values are presented as means ± SE. We used Prism 6 for Mac to analyze the data. The Mann–Whitney *U* test was used to evaluate the differences in characteristic between the DHE and non-DHE group such as age, height, weight, and BMI. The Mann–Whitney *U* test was used to evaluate the differences in parameters such as full mouth opening, Mallampati index, intergonion distance, mental–gonion distance, articulare–gonion distance, TMD, aperture angle, and cervical lordosis degree between the DHE and non-DHE groups. For statistical analysis of history of cervical spondylosis and radiotherapy, the Chi-square test was used.

## Results

### Patients

This study consisted of 51 patients, 37 in the non-DHE group and 14 in the DHE group. The patients had disease as shown in Table 1. One patient of DHE group underwent chemoradiotherapy owing to difficulty of hypopharyngeal exposure even with a curved laryngoscope. One patient of DHE group with cricopharyngeal dysfunction underwent transcervical cricopharyngeal myotomy. Characteristics of the two groups are summarized in Table 2. There were no significant differences between the non-DHE and DHE groups in age, height, weight, and BMI.

**Table 2:** Characteristic of non-DHE and DHE groups

	Non-DHE	DHE	*p*
Age	67.0 ± 2.2	68.0 ± 1.6	0.8465
Hight	165.5 ± 1.0	162.1 ± 1.5	0.0842
Weight	55.8 ± 1.9	56.6 ± 3.2	0.8143
Body mass index	20.3 ± 0.6	21.7 ± 1.5	0.6877

### Parameters evaluated

Comparisons of radiographic and cephalometry parameters between the non-DHE and DHE groups are summarized in Table 3. Regarding radiographic evaluation, there was a significant difference in cervical lordosis degree between the non-DHE and DHE groups (56.4 ± 1.9° vs. 38.7 ± 3.5°). On the other hand, there was no significant difference in full mouth opening between the non-DHE and DHE groups (48.9 ± 1.4 vs. 45.2 ± 2.9 mm). There was no significant difference in the Mallampati index between the non-DHE and DHE groups (1.8 ± 0.1 vs. 2.0 ± 0.2). In cephalometric evaluation, there were no significant differences between the non-DHE and DHE groups in intergonion distance (102.2 ± 0.9 vs. 103.9 ± 2.7 mm), mental–gonion distance (80.2 ± 0.9 vs. 76.4 ± 2.6 mm), articulare–gonion distance (63.9 ± 0.9 vs. 67.7 ± 1.5 mm), and TMD (79.4 ± 1.9 vs. 70.9 ± 4.9 mm).

**Table 3:** Comparisons of radiographic and cephalometry parameters between the non-DHE and DHE groups

	Non-DHE	DHE	*p*
Full mouth opening	48.9 ± 1.4	45.2 ± 2.9	0.1282
Mallampati index	1.8 ± 0.1	2 ± 0.2	0.3802
Intergonion distance	102.2 ± 0.9	103.9 ± 2.7	0.9023
Mental–gonion distance	80.2 ± 0.9	76.4 ± 2.6	0.2352
Articulare–gonion distance	63.9 ± 0.9	67.7 ± 1.5	0.08
Thyroid–mental distance	79.4 ± 1.9	70.9 ± 4.9	0.2209
Aperture angle	55.1 ± 1.7	50.0 ± 1.7	0.1533
Cervical lordosis degree	56.4 ± 1.9	38.7 ± 3.5	< 0.0001*
Cervical spondylosis	0.139	0.357	0.118
Past radiation	0.083	0.357	0.0304*

A higher proportion of patients with DHE had a history of radiotherapy compared with patients without DHE. However, no significant differences in the proportion of DHE patients with and without cervical spondylosis.

**Fig. 3:**
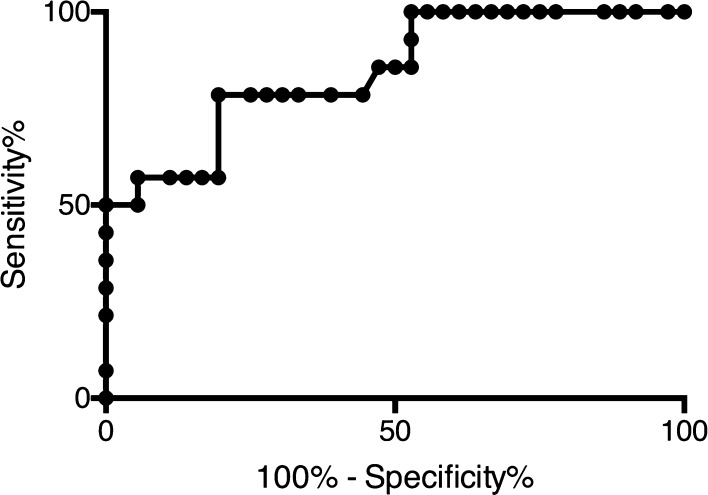
Receiver operating characteristic curve analysis of cervical lordosis degree. Cut-off value of 46.6° had 78.57% predicted sensitivity and 80.56% specificity for detecting the risk of DHE

Receiver operating characteristic curve analysis showed that with a cervical lordosis degree cut-off value of 46.6° had 78.57% predicted sensitivity and 80.56% specificity for detecting the risk of DHE (Fig. 3).

## Discussion

Endoscopic transoral procedures provide excellent oncologic outcomes while preserving speech and swallowing function [2]. Several studies have shown that TORS is a feasible, safe, and effective procedure for the resection of benign and malignant oropharyngeal, supraglottic, and hypopharyngeal tumors [2]. The high-resolution, magnified, three-dimensional view of the operative field provided by TORS allows for excellent visualization of the target anatomy [2]. Recently, alternative transoral surgical methods have been developed, such as TOVS and ELPS. Feasibility has been a major concern about transoral surgery. Therefore, ensuring visualization of the surgical field and sufficient working space is required. Limited access and bulky instruments are the main limitations, especially in TORS, where a moving mechanical arm is used [4]. Correct exposure of the lesion is a necessary condition for the success of the procedure. In TORS, good exposure means not only a complete view of the lesion but also sufficient space for the movement of the mechanical arm.

In TORS and TOVS, a straight plate such as the FKWO retractor is used to obtain laryngopharyngeal exposure. Using the FKWO retractor to expose the hypopharynx and supraglottis is laborious and patient-dependent. Important determinants of feasibility include patient suitability for anesthesia and sufficient access for target resection [2]. Therefore, Weinstein et al. advocated examination under anesthesia using a mouth gag to confirm feasibility and visualization.

Appropriate exposure of the lesion remains the key determinant of whether the procedure can be performed. If a lesion is difficult to approach and its boundaries are not clearly viewed, one risk not only a laborious surgical procedure, but also inadequate resection margins, a greater risk of damaging healthy structures, and causing unexpected bleeding [16]. Inadequate target exposure that could not permit TORS was reported to occur in 7–26% of cases [2, 17]. Identifying patients who are unsuitable is crucial to preventing unnecessary general anesthesia and minimizing the time and financial costs of abandoned TORS and conversion to open surgery [18].

Several parameters that could predict DLE have been reported: short neck, stiff and muscular neck, macroglossia, retrognathia, obesity, various anatomical measurements, and limited cervical spine extension [8, 19]. Furthermore, Arora et al. reported that anatomic biometric measurements using cadavers were useful in the decision-making process when assessing patient suitability for oropharyngeal and supraglottic TORS. There have been few reports regarding TORS for hypopharyngeal cancer in living subjects.

We evaluated the association between hypopharyngeal exposure and various parameters during transoral surgery. In this study, hypopharyngeal exposure was significantly correlated with the degree of cervical lordosis on radiography. This finding implies that patients with cervical spondylosis and restricted neck flexibility are likely to have inadequate hypopharyngeal exposure. A previous study showed that limited cervical extension is a predictor of DLE. However, those studies did not include objective evaluation. This study was the first to show the association between DHE and limited cervical extension with objective data based on radiographic assessment. However, a history of cervical spondylosis was not a significant predictor of DHE. This finding implied that cervical spondylosis was not identified before transoral surgery, even if present. Therefore, objective assessment such as the degree of cervical lordosis on radiography might be required. This study suggested that limited cervical extension is a risk factor for difficulty in gaining access to the pharynx.

In this study, a history of previous radiotherapy for head and neck cancer was significantly associated with DHE. Soft tissue fibrosis and sclerosis from radiotherapy influence laryngeal suspension [20]. Piazza et al. evaluated predictors of DLE by considering previous radiotherapy, micrognathia, trismus, and TMD collectively, and identified a clinical predictor score for DLE [20]. However, previous radiotherapy as single parameter for predicting DHE has not been evaluated. This study revealed a relationship between previous radiotherapy and hypopharyngeal exposure during transoral surgery based on objective data.

In this study, cephalometric parameters were not significantly associated with DHE. A previous study reported that micrognathia is a risk factor for DLE. This difference between that study and the present study may be the result of differences in race or study design. There have been few objective studies on the relationship between DLE and micrognathia. Our findings suggest that micrognathia is not a predictor of DHE.

In this study, general physical parameters such as height, weight, and BMI did not differ between the DHE and non-DHE groups, suggesting that the skeletal structure of patients in this study was similar. Further studies with patients with different physical constitutions will be required.

Some limitations of this study should be mentioned. We only used the FKWO retractor for hypopharyngeal exposure; no other gags such as Davis gag was used. However, visualization of the epiglottis was significantly better using the FK retractor compared to the Davis gag [18]. Thus, evaluation with the FKWO retractor in this study was appropriate.

This study suggested that patients with limited cervical extension and a history of radiotherapy might have DHE during transoral surgery. This is the first report to suggest a classification system for hypopharyngeal exposure during transoral surgery. A baseline for estimating DHE would be useful for otolaryngologists in the surgical decision-making process.
